# Antimicrobial use by WHO methodology at primary health care centers: a cross sectional study in Punjab, Pakistan

**DOI:** 10.1186/s12879-018-3407-z

**Published:** 2018-09-29

**Authors:** Muhammad Rehan Sarwar, Anum Saqib, Sadia Iftikhar, Tayyaba Sadiq

**Affiliations:** 1 Department of Pharmacy, The Islamia University of Bahawalpur, Punjab, Pakistan; 2 Akhtar Saeed College of Pharmaceutical Sciences, Lahore, Pakistan; 3 Department of Pharmacy, University of Sargodha, Punjab, Pakistan

**Keywords:** Antimicrobial resistance, Antimicrobial use, Primary health care centers

## Abstract

**Background:**

To investigate the antimicrobial (AM) use and prescribing patterns at primary health care centers (PHCCs) in Punjab, Pakistan.

**Methods:**

A cross-sectional study was designed according to the World Health Organization (WHO) methodology for AM usage from January, 2017 to June, 2017. Standard data collection forms designed by the WHO were used to collect the data from 32 PHCCs (16 rural healthcare centers (RHCs) and 16 basic health units (BHUs)) in Punjab province of Pakistan. PHCCs were randomly selected from 8 main cities. The study sample consisted of prescription records of 6400 outpatients (200 prescriptions records from each PHCC) and 800 inpatients (25 inpatient records from each PHCC). Data of the year 2016 were collected retrospectively by using systematic random sampling technique and analyzed through SPSS.

**Results:**

Among the hospital indicators, standard treatment guidelines (STGs) regarding the infectious diseases were not available in PHCCs. Number of days during which key AMs were out of stock was 12.1 days per month (range = 3.1–19.2). Out of total PHCC medicines costs, expenditures on AMs were 26.2% (range = 17.1–39.0). In case of prescribing indicators, the average number of AMs per prescription was 1.4 (range = 1.1–1.7), percentage of prescriptions prescribed with AMs was 81.5% (range = 68.9–89.1) and duration of AM treatment on average was 5.1 days per patient (range = 3.3–6.4). Average cost of prescribed AMs per patient was 1.3 USD (range = 0.6–4.3). The PHCCs prescribed a median of 5 (range = 3–9) types of AMs, including 10 (range = 5–15) individual agents. Out of 79.3% prescriptions of outpatients prescribed with AMs, only 16.4% were properly prescribed. Out of 100% prescriptions of inpatients prescribed with AMs, 12.1% were properly prescribed. Out of all the AM prescriptions 23.6% contained penicillins, 20.1% contained cephalosporins and 19.4% contained fluoroquinolones Metronidazole (18.0%), ciprofloxacin (16.5%) and co-amoxiclav (14.3%) were most commonly prescribed AMs.

**Conclusions:**

In PHCCs, AMs were prescribed more frequently. However large proportions of these prescriptions were inappropriate. Continuous education and training of medical staff and cost effective policies could play an important role in promotion of rational use of AMs.

**Electronic supplementary material:**

The online version of this article (10.1186/s12879-018-3407-z) contains supplementary material, which is available to authorized users.

## Background

Antimicrobial resistance (AMR) has emerged as a global issue and presents a major challenge regarding the treatment options of infectious diseases [1]. AMR result in decreased potency of antimicrobials (AMs) against causative microbes, unnecessary health costs and ultimately leads to failure of therapy. Various studies reported different factors causing the AMR but inappropriate or excessive prescribing of AMs and unnecessary usage of broad spectrum AMs were the main factors [2–4]. According to a study, AMs accounts for more than 30% of total hospital budget as one third to half of hospitalized patients receive at least one AM [5, 6]. Out of total use of AMs, 20–50% is not proper [2, 7, 8] leading to decreased quality of patient care, increased cost of therapy and prevalence of adverse drug effects [9].

AMR has emerged widely but developing countries are more affected by this issue due to lack of proper health care facilities and high rate of infections [10, 11]. In Pakistan, most of the physicians in government hospitals tend to prescribe those AMs that are not effective against majority of the causative microbes [12].

In Pakistan, hospitals are classified as primary, secondary and tertiary care hospitals. Primary health care centers (PHCCs) [RHCs = Rural health centers and BHUs = Basic health units] are small clinical set ups, providing limited medical facilities. Secondary care hospitals are county hospitals, while the tertiary care hospitals offer vast medical and clinical facilities and mostly located in big cities. Survey reports of AMs in secondary and tertiary care hospitals showed high prescribing rates of AMs as 48.9%, [13] 51.5%, [14] 52%, [15] and 52.4% [14] but there is unavailability of any data regarding prescribing trend of AMs in PHCCs with special reference to the World Health Organization (WHO) AM use indicators. Availability of this data is very important as majority of Pakistani population avails medical services at PHCCs, therefore understanding of AMs use in PHCCs and promotion of rational use of AMs is crucial.

The purpose of this study was to investigate the usage and prescribing patterns of AMs at 32 selected PHCCs in Punjab, Pakistan. AM usage was compared among RHCs and BHUs, outpatients and inpatients as well as in surgical and nonsurgical patients.

## Methods

### Study settings

The study was conducted in Punjab province of Pakistan, which have a population of 110,012,442 according to the census of 2017 [16]. The total PHCCs existing in 8 main cities of Punjab (Lahore, Faisalabad, Bahawalpur, Multan, Dera Ghazi Khan, Sargodha, Rawalpindi, and Gujranwala) are 128 (43 RHCs and 85 BHUs). 4 PHCCs (2 RHCs and 2BHUs) were randomly selected from the peripheries of each selected city so total 32 PHCCs (16 RHCs and 16 BHUs) were selected for this study. These selected PHCCs cover 14.3% of the total population of the Punjab i.e., 15,731,779. The characteristics of the selected PHCCs are summarized in Table 1.

**Table 1:** Characteristics of the selected primary health care centers, median (range)

Sr. no.	Characteristics	RHCs^a^(16)	BHUs^b^(16)	All PHCCs^c^(32)
1	Number of beds	9 (6–11)	4.5 (3–7)	6 (3–11)
2	Outpatients visit last year	36,658 (25,390 − 67,760)	21,312 (16,410 − 36,256)	29,297 (16,410 − 67,760)
3	Inpatients visit last year	1056 (783–1371)	413 (309–591)	756 (309–1371)
4	Prescribers/Medical officers	3.5 (3–5)	1.5 (1–3)	3 (1–5)
5	Nurses	6 (5–9)	3 (2–5)	4.5 (2–9)
6	Pharmacists/Dispensers	1.5 (1–3)	1 (1–2)	1 (1–3)
7	AM groups available	7 (5–9)	3 .5 (3–6)	5 (3–9)
8	AM agents available	11 (9–15)	7 (5–11)	10 (5–15)

^a^Rural Health Centers; ^b^ Basic Health Units; ^c^Primary Health Care Centers; *AM* Antimicrobial

### Study design and outcome variables

It was an observational and cross-sectional study, designed according to the objectives of the study. The outcome indicators were related to two general areas of AMs usage i.e., hospital indicators and prescribing indicators. The AMs usage patterns in terms of frequency and percentages were also determined. The Anatomical Therapeutic Chemical (ATC) classification system [17] was used for the coding of AMs.

### Study inclusion / exclusion criteria

The inclusion criteria were based on the time period of study, health status (acute and chronic illness), annual bulk purchase data and duration of treatment, whereas records of pre-existing infections, multiple and local purchase data were excluded from the study (Additional file 1).

### Sampling and data collection

The standard indicator forms were used to collect the data. Reliability of the data was assured by adhering to WHO guidelines and methods [10, 18]. The data were collected from January, 2017 to June, 2017. Two investigators (Pharm.D students) were assigned to each PHCC; all investigators received the same training prior to the survey for the collection and validation of data. During the survey, one investigator filled out the investigational forms while the other reviewed the data. All data were checked for completeness and logicality.

The data of hospital indicators were collected over a period of 1 year (January, 2016 to December, 2016). Most recent copies of formulary list/essential medicines list (FL/EML), key AMs and STGs were obtained from the Pharmacy Departments of the PHCCs.

Out of the total 921,311 outpatient prescriptions and 25,184 inpatient prescriptions, a retrospective selection of 6400 outpatient and 800 inpatient prescription records (200 outpatients and 25 inpatients per PHCC) was made over a period of 1 year i.e., from January, 2016 to December, 2016. To minimize the selection bias, prescription records written for the prescribed time period in each of the selected PHCCs were divided into four quarters and from each quarter 50 outpatient and 6 inpatient prescription records were selected by using systematic random sampling technique [19]. From these prescription records, the WHO prescribing indicators and prescribing patterns of AMs were determined.

AMs prescription was judged according to “WHO prescribing and hospital indicators” [18, 20], “Infectious Diseases Society of Pakistan (IDSP) guidelines for AMs use” [21] and “American Thoracic Society Consensus Guidelines on the Management of Community-Acquired Pneumonia in Adults” [22]. The expert opinion was taken from the local team of consultant pharmacists, microbiologists and the physicians having specialization in infectious diseases. The discrepancy between investigators regarding assessment of appropriateness of AMs therapy was also resolved by consulting the aforementioned team of consultants. The AMs usage was considered to be proper (correct decision) if it contained the standard treatment regimen and duration that was indicated for the patient’s symptoms of infection or prophylaxis. AMs usage was considered improper if clinical condition of the patient did not justify use of AMs for either treatment or prophylaxis (incorrect/missing data and incorrect decision) [21, 23, 24].

### Data analysis

Statistical Package for Social Sciences (IBM Corp. Released 2012. IBM SPSS Statistics for Windows Version 21.0. Armonk, NY: IBM Corp.) and Microsoft Excel (MS Office 2010) were used for data analysis. Kolmogorov-Smirnov and Shapiro-Wilks tests were carried out to test the normality of the data. Median was used, when the data showed non-normal distribution. Independent Samples Mann-Whitney U Test was employed to assess the difference among the PHCCs, and a *p-value* < 0.05 was used for statistical significance of differences.

## Results

### Hospital indicators

In all PHCCs, the Drug and Therapeutic Committee (DTC) was working on regular basis and a FL/EML was available that contains 15 generics of AMs. 15 AMs listed in FL/EML were available in 22 different dosage forms. Out of these 22 dosage forms, an average of 15 dosage forms (68.2%, range = 7–22) were available in the stock at the day of data collection. Average number of days during which a set of key AMs was out of stock in all PHCCs was found to be 12.1 (3.1–19.2) days per month for the 15 AMs. The difference among the PHCCs was statistically significant for 6─10 hospital indicators (Table 2). The results of the hospital indicators are summarized in Table 2.

**Table 2:** WHO hospital indicators at the selected primary health care centers

Sr. No.	Indicator	RHCs^g^(*n* = 16)	BHUs ^h^(*n* = 16)	All PHCCs^i^(*n* = 32)	*p-value*
1	Existence of DTC^b^, %	100	100	100	-------^a^
2	Availability of STGs^c^ for infectious diseases, %	0.0	0.0	0.0	-------^a^
3	Availability of FL/EML^d^_,_ %	100	100	100	-------^a^
4	Number of AMs on the FL/EML	15	15	15	-------^a^
5	Medicines identified by INN^e^_,_ %	100	100	100	-------^a^
6	Key AMs in stock, %	73.8 (60–100)	47.9 (33.3–73.3)	60.8 (33.3–100)	< 0.001
7	No. of days/month that a set of key AMs is out of stock, mean (range)	7.4 (3.1–9.7)	15.4 (8.7–19.2)	12.1 (3.1–19.2)	< 0.001
8	Total number of patients discharged per PHCC during the last calendar year	1056 (783–1371)	413 (309–591)	756 (309–1371)	< 0.001
9	Surgeries performed during the last calendar year	Major = 47.5 (29–93)	Major = 17.5 (11–33)	Major = 29.5 (11–93)	< 0.001
		Minor = 219 (165–303)	Minor = 134.5 (104–207)	Minor = 189 (104–303)	
10	Expenditure on AMs^f^ per total PHCC medicine costs, %	23.2 (17.1–29.5)	31.3 (21.4–39.0)	26.2 (17.1–39.0)	< 0.001

^a^Mann-Whitney U Test was not applied for these indicators as there was no variation in their values, ^b^Drug and therapeutic committee; ^c^Standard treatment guidelines; ^d^Formulary list/essential medicines list; ^e^International non-proprietary names; ^f^Annual bulk purchase data only; ^g^Rural health centers; ^h^Basic health units; ^i^Primary health care centers

### Prescribing indicators

The percentage of prescriptions with at least one AMs prescribed was 81.5% (range = 68.9–89.1) and from these patients, average number of AMs per prescription was 1.4 (range = 1.1–1.7). The difference among the PHCCs was statistically significant for 1st, 2nd, 4th, and 5th prescribing indicator (Table 3). Results regarding prescribing indicators in the selected PHCCs are summarized in Table 3.

**Table 3:** WHO prescribing indicators at the selected primary health care centers

Sr. No.	Indicator	RHCs^e^(*n* = 16)	BHUs^f^(*n* = 16)	All PHCCs^g^(*n* = 32)	*p-value*
1	Prescriptions with one or more AMs^b^, % (range)	79.3 (68.9–86.2)	83.8 (71.4–89.1)	81.5 (68.9–89.1)	< 0.001
2	AMs prescribed per prescription, mean (range)	1.3 (1.1–1.5)	1.5 (1.2–1.7)	1.4 (1.1–1.7)	< 0.001
3	AMs prescribed from FL/EML^h^, %	100	100	100	-------^a^
4	Cost (USD^c^) of AMs prescribed per prescription, mean (range)	1.5 (0.6–4.3)	0.9 (0.6–3.7)	1.3 (0.6–4.3)	< 0.001
5	Duration of prescribed AMs (days), mean (range)	5.2 (3.3–6.4)	4.9 (3.7–5.8)	5.1 (3.3–6.4)	< 0.001
6	Pneumonia patients who received AMs, %	100	100	100	-------^a^
7	Patients who received AMs for Pneumonia in accordance with clinical guidelines^i^, %	0.0	0.0	0.0	-------^a^
8	AMs prescribed by INN^d^, %	100	100	100	-------^a^

^a^Mann-Whitney U Test was not applied for these indicators as there was no variation in their values, ^b^Antimicrobials; ^c^US Dollars;^d^International non-proprietary name; ^e^Rural health centers; ^f^Basic health Units; ^g^Primary health care centers; ^h^Formulary list/Essential medicines list; ^i^Infectious Diseases Society of America/American Thoracic Society Consensus Guidelines on the Management of Community-Acquired Pneumonia in Adults (https://www.thoracic.org/statements/resources/mtpi/idsaats-cap.pdf); ^d^International non-proprietary names

### Antimicrobial use in outpatients and inpatients

In all PHCCs 79.2% (76.7% in RHCs and 81.8% in BHUs) of outpatient prescriptions contained one or more AMs, out of which 22.1% in RHCs, 11.9% in BHUs and 16.4% in all PHCCs were proper. 100% of inpatient prescriptions contained one or more AMs, out of which 14.3% in RHCs, 11.6% in BHUs and 12.1% in all PHCCs were proper. Out of the 436 surgical inpatient prescriptions, 13.1% in RHCs, 11.7% in BHUs and 11.9% in all PHCCs were proper and of the 364 nonsurgical inpatient prescriptions, 15.1% in RHCs, 11.3% in BHUs and 12.9% in all PHCCs were proper (Table 4).

**Table 4:** Antimicrobial use in Outpatients and Inpatients

Indicator	RHCs^b^ (*n* = 3600)	BHUs^c^ (*n* = 3600)	All PHCCs (*n* = 7200)
Outpatients (*n* = 6400)			
1. Prescriptions with one or more AMs^a^, %	76.7	81.8	79.2
2. AMs prescribed per prescription, mean (SD)	1.0 (0.23)	1.3 (0.41)	1.2 (0.31)
3. % of prescriptions with injected AMs	31.6	42.7	36.8
4. Proper AMs use, %	22.1	11.9	16.4
Inpatients all (*n* = 800)			
1. Prescriptions with one or more AMs^a^, %	100	100	100
2. AMs prescribed per prescription, mean (SD)	2.9 (0.11)	3.1 (0.13)	3.0 (0.12)
3. % of prescriptions with injected AMs	40.9	44.7	43.6
4. Proper AMs use, %	14.3	11.6	12.1
A. Surgical (*n* = 436)			
1. Prescriptions with one or more AMs^a^, %	100	100	100
2. AMs prescribed per prescription, mean (SD)	2.9 (0.11)	3.1 (0.13)	3.0 (0.12)
3. Proper AMs use, %	13.1	11.7	11.9
B. Nonsurgical (*n* = 364)			
1. Prescriptions with one or more AMs^a^, %	100	100	100
2. AMs prescribed per prescription, mean (SD)	2.9 (0.11)	3.1 (0.13)	3.0 (0.12)
3. Proper AMs use, %	15.1	11.3	12.9

^a^Antimicrobials, ^b^Rural Health Centers, ^c^Basic Health Units

### Prescribing patterns of antimicrobials

81.5% (*n* = 5868) prescriptions were prescribed with a total of 8236 AMs especially in gastrointestinal tract (GIT) infections (16.4%, *n* = 1182), urinary tract infections (UTIs) (13%, *n* = 937) and acute bronchitis (11%, *n* = 801) (Additional file 2). Overall Penicillins (23.6%, *n* = 1944), cephalosporins (20.1%, *n* = 1658) and fluoroquinolones (19.4%, *n* = 1594) were the most frequently prescribed AM classes at the selected PHCCs (Additional file 3). But in comparison of BHUs with RHCs, penicillins (25.3%, *n* = 1143) and fluoroquinolones (22.8%, *n* = 1030) were mostly prescribed in BHUs while cephalosporins (22.3%, *n* = 829) in RHCs. Metronidazole (18.0%, *n* = 1480), ciprofloxacin (16.5%, *n* = 1357) and co-amoxiclav (a combination of amoxicillin and clavulanic acid) (14.3%, *n* = 1176) were the most frequently prescribed AMs at the selected PHCCs (Table 5).

**Table 5:** Antimicrobial agents being prescribed at the selected primary health care centers

Sr. No.	ATC^a^ Code	Antimicrobial name	RHCs ^b^(*n* = 3711)	BHUs ^c^(*n* = 4525)	Outpatients (*n* = 5853)	Inpatients (*n* = 2383)	All (*N* = 8236)
1	J01AA02	Doxycycline	131 (3.5)	97 (2.1)	228 (3.9)	–	228 (2.8)
2	J01CA01	Ampicillin	304 (8.2)	464 (10.3)	768 (13.1)	–	768 (9.3)
3	J01CR02	Amoxiclav	497 (13.4)	679 (15.0)	863 (14.7)	313 (13.1)	1176 (14.3)
4	J01DB09	Cephradine	207 (5.6)	109 (2.4)	297 (5.1)	19 (0.8)	316 (3.8)
5	J01DD01	Cefotaxime	134 (3.6)	77 (1.7)	89 (1.5)	122 (5.1)	211 (2.6)
6	J01DD04	Ceftriaxone	488 (13.2)	643 (14.2)	522 (8.9)	609 (25.6)	1131 (13.7)
7	J01EE01	Cotrimoxazole	53 (1.4)	31 (0.7)	69 (1.2)	15 (0.6)	84 (1.0)
8	J01FA09	Clarithromycin	219 (5.9)	242 (5.3)	418 (7.1)	43 (1.8)	461 (5.6)
9	J01FF01	Clindamycin	123 (3.3)	91 (2.0)	214 (3.7)	–	214 (2.6)
10	J01FF02	Lincomycin	212 (5.7)	197 (4.4)	409 (7.0)	–	409 (5.0)
11	J01GB03	Gentamicin	79 (2.1)	42 (0.9)	–	121 (5.1)	121 (1.5)
12	J01GB06	Amikacin	29 (0.8)	14 (0.3)	25 (0.4)	18 (0.8)	43 (0.5)
13	J01MA02	Ciprofloxacin	451 (12.2)	906 (20.0)	1163 (19.9)	194 (8.1)	1357 (16.5)
14	J01MA14	Moxifloxacin	113 (3.0)	124 (2.7)	108 (1.8)	129 (5.4)	237 (2.9)
15	J01XD01	Metronidazole	671 (18.1)	809 (17.9)	680 (11.6)	800 (33.6)	1480 (18.0)

^a^Anatomical therapeutic chemical classification system. ^b^Rural health centers; ^c^Basic health units

## Discussion

Irrational use of AMs is spreading over the globe as an international public health threat. But this problem is more prevalent in the developing countries due to scarcity of resources, higher rates of infections and less availability of medical facilities [10, 11]. This study targeted 32 PHCCs in which the practices regarding AMs usage had been the core objective of investigation. Results of this study could be used by policy makers to assess and improve AMs usage along with the promotion of rational use of AMs in Pakistan. AMs usage was very high in inpatients as well as in outpatients. In prescriptions of patients, there was evidence of AMs usage without clear indications and observance of many errors. Moreover, unnecessary prescribing pattern of the broad spectrum AMs, multiple combinations, prolonged usage and intravenous administration of AMs were also observed in this study.

### Hospital indicators

The presence of STGs and FL/EML in health care facilities represents provision of good quality patient care and promotion of rational use of medicines [10]. All PHCCs contained EML that had 15 generic AMs. The Drug and Therapeutic Committee (DTC) functions in an ongoing basis in the study settings and consequently the EML was revised, updated and approved annually by the administration of the PHCCs. None of the PHCCs had STGs for infectious diseases. Due to this reason, prescribers do not have any STGs for prescribing and this obstacles rational prescribing of AMs. Ultimately quality of patient care was compromised [10].

Along with the provision of STGs, there must be sufficient availability of key AMs at all the time in PHCCs. There was 100% availability of key AMs on the day of study. This result is comparable to a study from Ethiopia that reported 90.1% availability of key AMs in the stock [25]. Stock maintenance of health care facility can be determined by the number of days during which there is an unavailability of AMs. Resulting value of this indicator was 12.1 days per month for 15 key AMs, making it higher than Afghanistan (8.7 days per month for 15 key AMs) [26] and Ethiopia (15–45 days over a 12-months period) [25]. Findings revealed that 60.8% key AMs were in stock. Unavailability of key AMs could be the reason for; patients not treated with drug of choice, financial burden and treatment failure. This noncompliance may lead to an increase in rates of morbidity and mortality [10].

Due to irrational and excessive use of AMs, expenditures on single class of drugs are on boom. *Indicator 5* records the cost of antimicrobials and demonstrates it as percentage of total hospital medicines costs. This study showed that expenditure on AMs (annual bulk purchase data only) was 26.2% of the total annual budget (Table 3). This is the cause of financial burden; hence patient treatment protocols are compromised.

### Prescribing indicators

Extent of AMs prescribing in health care facilities was determined by the *Indicator 6.* In this study the percentage of prescriptions containing AMs was 81.5% (Table 4). This value was lower than that reported from Afghanistan (90%) [26] and Nepal (93%) [27], whereas higher than Ethiopia (79.8%) [25], Thailand (44%) [28], Tanzania (35.4%) [29], Brazil (28.8%) [30] and Bangladesh (25%) [31]. Inpatients may receive more than one AMs depending upon health condition but could further lead to irrational use, inappropriate combination therapy and unnecessary changes in dosage regimen [10]. In this study, the average number of AMs per prescription was 1.4 and this value was lower than that reported in Ethiopia (1.2) [25] and higher than Afghanistan (1.7) [26] and Nepal (2.4) [27]. Treatment duration with AMs for infectious diseases is 7–10 days but some advance infectious diseases require longer duration such as osteomyelitis and meningitis [10]. Average duration of prescribed AMs treatment in PHCCs was 5.1 days and comparable to study from Afghanistan that reported it to be 5 days [26].

The WHO recommended prescribing of medicines by their generic names. Study showed that percentage of AMs prescribed by generic names was100%. This value was higher than the studies conducted in Thailand (87%) [28] and Afghanistan (88%) [26]. Prescribing of AMs by the brand names may lead to increase risks of morbidity and mortality along with financial burdens on health care budgets [32].

AMs may contribute towards financial burden due to irrational prescribing as they contribute more than 30% of the total hospital budget [5]. The results of current study revealed that the average cost of AMs prescribed per patient was 1.3 USD. Though this cost is 5 folds less as compared to the cost of AMs prescribed per patient in Nigeria but still it poses huge financial encumbrance on healthcare sector of Pakistan [33]. In public sector hospitals of Pakistan, government is solely responsible to bear health care costs and unfortunately no proper health insurance schemes have been developed till date. This is the main reason of limited stock and unavailability of AMs and essential medicines in government hospitals.

STGs are the main pillar to be relied upon for the promotion of rational prescribing of AMs. It is also necessary to follow the STGs for proper treatment of pneumonia and other infections [26]. Unavailability of STGs is the leading cause of irrational prescribing, prolong hospital stay and increase cost of therapy. The adherence of prescribers to the hospital’s STGs depends upon two factors; prescribing only those AMs listed in STGs; prescribing doses mentioned in the STGs [10] Results of the current study concluded that none of the pneumonia patients received AMs in accordance with the WHO AM use indicators, IDSP guidelines and American Thoracic Society Consensus Guidelines on the Community-acquired Pneumonia in Adults. Due to the absence of STGs in PHCCs previously published guidelines mentioned in the literature were used as a reference [22]. These results were in accordance with a similar study from Afghanistan in which none of the patients received treatment in accordance to STGs [26].

### Prescribing patterns of antimicrobials

According to a study 35–60% of the patients were prescribed with AMs and less than 20% were prescribed properly [34]. In current study, 81.5% of the prescriptions had one or more AMs especially in GIT infections (16.4%), UTI (13%) and acute bronchitis (11%). GIT infections (bacterial and viral origin) are prevailing in Pakistan because of unhygienic sanitary conditions, contaminated water and edibles. AMs, especially fluoroquinolones, are frequently prescribed in GIT infections (bacterial origin) to lessen the severity of infection and to minimize other symptoms [35]. Patients suffering from UTI are frequently prescribed with cephalosporins and penicillins [36]. Acute bronchitis is mostly caused by bacteria and viruses and AMs are prescribed to reduce the duration of cough and other symptoms [37].

Rate of AMs usage in PHCCs in Pakistan is comparable to China (75.9%) [1], India (78%) [38], Indonesia (84%) [39] and Jordan (85%) [40]. AMs usage in Pakistan was exceeded as compared to developed countries like the United States (63.7%) [41] and Sweden (30%) [42].

In Pakistan, India and China the most commonly prescribed AMs were penicillins, fluoroquinolones (in BHUs) and cephalosporins (in RHCs). Contrary to European countries, most commonly used AMs for outpatients were tetracyclines, benzylpencillins and sulfonamides [38, 42]. The results of the current study revealed that most frequently prescribed AMs included metronidazole (18.0%), ciprofloxacin (16.5%) and co-amoxiclav (14.3%). Higher prescribing rates of AMs may be due to better clinical outcomes, stock availability or excessive marketing campaigns. A study performed in Ethiopia revealed that the most frequently prescribed AMs were penicillin G (28.4%), ceftriaxone (24.9%) and cloxacillin (12.8%) [25]. Indian study showed that the highest prescribed AMs were levofloxacin (25.8%), metronidazole (14.8%) and ceftriaxone (12.7%). Treatment duration for most of the infections extends to few days but complicated and severe infectious cases may require multiple AMs usage for extended period of time. [43–45] These multiple AMs treatments are usually considered to provide broad AMs spectrum [46].

This study revealed that 12.1% of inpatients and 16.4% of outpatients were prescribed properly. Contributing factors towards inappropriate use of AMs in PHCCs in Pakistan could be lack of medical facilities and health experts. In both the RHCs and BHUs, medical staff was not skilled properly but in RHCs physicians having specialization degrees are working as compared to BHUs which is under the supervision of medical officer (bachelor’s degree holder). This might be the reason that our findings showed proper usage more in RHCs than in BHUs. Medical facilities are more directed towards secondary and tertiary care hospitals in cities further limiting exposure and training opportunities of PHCCs staff. Lack of proper medical facilities and unavailability of STGs lead to irrational prescribing of broad-spectrum AMs, use of multiple AMs in different combinations, excessive AMs use in prophylaxis for surgical patients and longer duration of therapy. The findings of the current study could be used as a baseline for further follow-up for quality of AMs usage in the future. Furthermore these findings will also help the policy makers to implement appropriate interventions designed to improve rational use of AMs at PHCCs of Pakistan in specific and globally at large.

### Strengths and limitations of the study

To the author’s best knowledge, there is no previously published data available (neither in Asian region nor in African region) on the WHO AM use indicators with special focus on current practices in PHCCs regarding AM use. This study enlightens the path of future researchers, policy makers and stakeholders in Pakistan.

This study has some limitations. First, the daily doses of AMs were not measured. Second, the study was conducted in a single province of Pakistan so results could not be generalizable to entire country. However, the condition of healthcare sector is similar across the country and similar findings are expected nationwide. Last, the reasons accountable for irrational prescribing were not inquired. Future studies should focus on these aspects.

## Conclusions

The results of the current study highlighted irrational AMs utilization patterns concerning availability of STGs, AMs stock shortage days, and percentage of AMs prescribing. Substantial improper AMs use occurs in PHCCs in Pakistan, probably because of the inadequate education and skill levels of professional staff, and weak oversight. To overcome these shortcomings, DTC should develop and implement the STGs for infectious diseases at the PHCCs. Pharmacists (having specialty in infectious diseases) should be appointed because these professionals can play critical role in improving the current scenario by developing and implementing surveillance system in the hospital; searching and providing data about the most common strains of microbes in Punjab and promoting rational prescribing trend among prescribers on continuous basis. Furthermore, prime focus must be given to prescriber’s training regarding rational use of AMs and the development of cost-effective interventions by the health authorities and the policy makers respectively.

## Additional files

Additional file 1: Study inclusion/exclusion criteria. (DOCX 16 kb)

Additional file 2: Conditions in which antimicrobials being prescribed at the selected primary health care centers. (DOCX 16 kb)

Additional file 3: Antimicrobial classes being prescribed at the selected primary health care centers. (DOCX 15 kb)

## Abbreviations

AMR: Antimicrobial Resistance
AMs: Antimicrobials
ATC: Anatomical therapeutic chemical classification system
BHU: Basic health units
FL/EML: Formulary list/Essential medicines list
PHCCs: Primary health care centers
RHC: Rural health centers
SPSS: Statistical packages for social sciences
STGs: Standard treatment guidelines
WHO: World Health Organization
