# Supplementary material for: Antimicrobial use by WHO methodology at primary health care centers: a cross sectional study in Punjab, Pakistan
Source: BMC Infect Dis. 2018 Sep 29;18:492. doi: 10.1186/s12879-018-3407-z (PMC6162939; doi:10.1186/s12879-018-3407-z)
Supplement: Supplementary file 1 — Study inclusion/exclusion criteria. (DOCX 16 kb) [file 12879_2018_3407_MOESM1_ESM.docx]

**Study inclusion/exclusion criteria**

| **Sr. No.** | **Indicator** | **Inclusion/exclusion criteria** |
| --- | --- | --- |
| 1 | All | Inpatient and outpatient prescription records of time period from January, 2016 to December, 2016, were included in the study. |
| 2 | All | The sample was limited to the encounters of acute and chronic illnesses along with health conditions and age of the patients. |
| 3 | All | Referral, tuberculosis, human immunodeficiency virus and vaccination cases were excluded from the study. |
| 4 | *Indicator 5* | Only the annual bulk purchase costs of AMs were included in the study because multiple and local purchase data were not readily available. |
| 5 | *Indicator 10* | This indicator includes duration of treatment with AMs and did not include prophylaxis AMs treatments. |
| 6 | *Indicators 11, 12* | The prescription records with pre-existing infections were excluded from the study. |
| 7 | *Indicator 14* | Generic or international non-proprietary names (INN) were those, as identified in the WHO list of INN[17]. |

**AMs**: Antimicrobials; **WHO**: World Health Organization; **INN**: International non-proprietary names
