# Supplementary material for: Antimicrobial use by WHO methodology at primary health care centers: a cross sectional study in Punjab, Pakistan
Source: BMC Infect Dis. 2018 Sep 29;18:492. doi: 10.1186/s12879-018-3407-z (PMC6162939; doi:10.1186/s12879-018-3407-z)
Supplement: Supplementary file 2 — Conditions in which antimicrobials being prescribed at the selected primary health care centers. (DOCX 16 kb) [file 12879_2018_3407_MOESM2_ESM.docx]

**Conditions in which antimicrobials being prescribed at the selected primary health care centers**

| **Condition** | **RHCs ^¶^**  **(n = 3,600)** | **BHUs ^‡^**  **(n = 3,600)** | **Outpatients (n = 6,400)** | **Inpatients**  **(n = 800)** | **All**  **(N = 7,200)** |
| --- | --- | --- | --- | --- | --- |
| Acute bronchitis | 376 (10.4) | 425 (11.8) | 801 (12.5) | ----- | **801 (11.1)** |
| Cesarean section | 287 (8.0) | 149 (4.1) | ----- | 436 (54.5) | 436 (6.1) |
| Common colds | 359 (10.0) | 397 (11.0) | 756 (11.8) | ----- | 756 (10.5) |
| COPD | 130 (3.6) | 89 (2.5) | 165 (2.6) | 54 (6.8) | 219 (3.0) |
| GIT infections* | 608 (16.9) | 574 (15.9) | 1015 (15.9) | 167 (20.9) | **1182 (16.4)** |
| Otitis Media | 143 (4.0) | 203 (5.6) | 346 (5.4) | ----- | 346 (4.8) |
| PUD | 191 (5.3) | 126 (3.5) | 317 (5.0) | ----- | 317 (4.4) |
| Pharyngitis | 266 (7.4) | 387 (10.8) | 653 (10.2) | ----- | 653 (9.1) |
| Pneumonia | 125 (3.5) | 94 (2.6) | 132 (2.1) | 87 (10.9) | 219 (3.0) |
| Skin infections** | 260 (7.2) | 377 (10.5) | 637 (10.0) | ----- | 637 (8.8) |
| Typhoid fever | 366 (10.2) | 331 (9.2) | 641 (10.0) | 56 (7.0) | 697 (9.7) |
| UTI | 489 (13.6) | 448 (12.4) | 937 (14.6) | ----- | **937 (13.0)** |

* Diarrhea, gastroenteritis, abdominal cramps;**Impetigo, folliculitis, cellulitis; ^¶^ Rural health centers; ‡ Basic health units; **COPD:** Chronic obstructive pulmonary disease; **GIT:** Gastrointestinal tract; **PUD:** Peptic ulcer disease; **UTI:** Urinary tract infection
