# Supplementary material for: Antimicrobial use by WHO methodology at primary health care centers: a cross sectional study in Punjab, Pakistan
Source: BMC Infect Dis. 2018 Sep 29;18:492. doi: 10.1186/s12879-018-3407-z (PMC6162939; doi:10.1186/s12879-018-3407-z)
Supplement: Supplementary file 3 — Antimicrobial classes being prescribed at the selected primary health care centers. (DOCX 15 kb) [file 12879_2018_3407_MOESM3_ESM.docx]

**Antimicrobial classes being prescribed at the selected primary health care centers**

| **Sr. No.** | **ATC^*^ Code** | **Antimicrobial class** | **RHCs^¶^**  **(n = 3,711)** | **BHUs ^‡^**  **(n = 4,525)** | **Outpatients**  **(n = 5,853)** | **Inpatients**  **(n = 2,383)** | **All (N = 8,236)** |
| --- | --- | --- | --- | --- | --- | --- | --- |
| 1 | J01AA | Tetracyclines | 131 (3.5) | 97 (2.1) | 228 (3.9) | ----- | 228 (2.8) |
| 2 | J01CA/R | Penicillins | 801 (21.6) | 1,143 (25.3) | 1,631 (27.9) | 313 (13.1) | **1,944 (23.6)** |
| 3 | J01DB/D | Cephalosporins | 829 (22.3) | 829 (18.3) | 908 (15.5) | 750 (31.5) | **1,658 (20.1)** |
| 4 | J01EE | Sulfonamides | 53 (1.4) | 31 (0.7) | 69 (1.2) | 15 (0.6) | 84 (1.0) |
| 5 | J01FA | Macrolides | 219 (5.9) | 242 (5.3) | 418 (7.1) | 43 (1.8) | 461 (5.6) |
| 6 | J01FF | Lincosamides | 335 (9.0) | 288 (6.4) | 623 (10.6) | ----- | 623 (7.6) |
| 7 | J01JB | Aminoglycosides | 108 (2.9) | 56 (1.2) | 25 (0.4) | 139 (5.8) | 164 (2.0) |
| 8 | J01MA | Fluoroquinolones | 564 (15.2) | 1,030 (22.8) | 1,271 (21.7) | 323 (13.6) | **1,594 (19.4)** |
| 9 | J01XD | Imidazoles | 671 (18.1) | 809 (17.9) | 680 (11.6) | 800 (33.6) | 1,480 (18.0) |

**^*^**Anatomical therapeutic chemical classification system; ^¶^ Rural health centers; ‡ Basic health units
